# Non-invasive detection of lymphoma with circulating tumor DNA features and protein tumor markers

**DOI:** 10.3389/fonc.2024.1341997

**Published:** 2024-01-19

**Authors:** Yu Chang, Shiyong Li, Zhiming Li, Xinhua Wang, Fangyuan Chang, Shuaipeng Geng, Dandan Zhu, Guolin Zhong, Wei Wu, Yinyin Chang, Shichun Tu, Mao Mao

**Affiliations:** ^1^Department of Oncology, The First Affiliated Hospital of Zhengzhou University, Zhengzhou, China; ^2^Research and Development, SeekIn Inc, Shenzhen, China; ^3^Department of Internal Medicine, Sun Yat-sen University Cancer Center, Guangzhou, China; ^4^Clinical Laboratories, Shenyou Bio, Zhengzhou, China; ^5^Yonsei Song-Dang Institute for Cancer Research, Yonsei University, Seoul, Republic of Korea

**Keywords:** lymphoma, early diagnosis, copy number aberration, fragment size, end motif, EBV

## Abstract

**Background:**

According to GLOBOCAN 2020, lymphoma ranked as the 9^th^ most common cancer and the 12^th^ leading cause of cancer-related deaths worldwide. Traditional diagnostic methods rely on the invasive excisional lymph node biopsy, which is an invasive approach with some limitations. Most lymphoma patients are diagnosed at an advanced stage since they are asymptomatic at the beginning, which has significantly impacted treatment efficacy and prognosis of the disease.

**Method:**

This study assessed the performance and utility of a newly developed blood-based assay (SeekInCare) for lymphoma early detection. SeekInCare utilized protein tumor markers and a comprehensive set of cancer-associated genomic features, including copy number aberration (CNA), fragment size (FS), end motif, and lymphoma-related virus, which were profiled by shallow WGS of cfDNA.

**Results:**

Protein marker CA125 could be used for lymphoma detection independent of gender, and the sensitivity was 27.8% at specificity of 98.0%. After integrating these multi-dimensional features, 77.8% sensitivity was achieved at specificity of 98.0%, while its NPV and PPV were both more than 92% for lymphoma detection. The sensitivity of early-stage (I-II) lymphoma was up to 51.3% (47.4% and 55.0% for stage I and II respectively). After 2 cycles of treatment, the molecular response of SeekInCare was correlated with the clinical outcome.

**Conclusion:**

In summary, a blood-based assay can be an alternative to detect lymphoma with adequate performance. This approach becomes particularly valuable in cases where obtaining tissue biopsy is difficult to obtain or inconclusive.

## 1 Introduction

Lymphoma is a type of common cancer worldwide, including a large group of lymphoid hematopoietic malignancies, which can be further classified as Non-Hodgkin lymphoma (NHL) and Hodgkin lymphoma (HL) two major types (1). Lymphoma was the ninth most common cancer and the 12^th^ leading cause of cancer death according to GLOBOCAN 2020 (2). As counted in 2016, the incidence and mortality of lymphoma were 6.5 and 3.73 per 100,000, respectively, and lymphoma is considered as one of the top three cancer incidences in children for boys in China (3). Lymphoma is a diverse group of malignancies that originated from B, T, and NK cells, which includes more than 30 unique subtypes (4, 5). Currently, diagnosis and classification of lymphoma was mainly based on excisional or punctured tissues by immunohistochemistry and *in situ* hybridization (4, 6, 7). However, the invasive operation has significant limitations, including potential procedural risks and inter- and intra-tumor heterogeneity (8–10). Especially for patients with lesions that are difficult to reach (e.g., those in brain; deep lymph nodes in the thoracic cavity or abdomen).

In the past few years, an emerging technique named liquid biopsy could potentially improve these limitations. Analysis of circulating cell-free DNA (cfDNA) is the leading non-invasive liquid biopsy approach and has been extensively utilized for cancer early diagnosis (11–13), through capturing early cancer signals from circulating tumor DNA (ctDNA). Although the majority of cfDNA was often not of cancerous origin, technical advances in next-generation sequencing (NGS), together with advanced computational methods, allowing for identifying tumor specific alterations or features, such as single nucleotide variations (11, 14), copy number aberrations (CNA) (15–19) and fragment-omics (16, 20–22), and epigenetic changes (23–28). With regard to lymphoma, liquid biopsy has been used for classification of lymphoma subtype, assessment of tumor burden as a prognostic biomarker, and detection of molecular response to therapy (29–31). All of them were based on the PCR or NGS target region assays to detect lymphoma specific mutations. Most lymphoma patients did not have hotspot mutations, and most mutations detected from cfDNA came from hematopoietic stem cells and accumulated over time, which resulted in false positive prediction. However, CNAs were rarely detected in healthy individuals.

Here, we have developed a blood-based multi-omics assay (named SeekInCare) (18). It utilized traditional protein tumor markers (PTMs) and cancer genomic hallmarks including CNA, fragment size (FS), end motif, and cancer-related virus which were evaluated by shallow whole-genome sequencing with the aim to calculate the cancer risk score (CRS). SeekInCare making full use of the common cancer genomic features, CNA, FS, end motif, and a panel of seven protein markers, is not limited to a particular cancer type and has been reported in several cancers (16, 20–22). Therefore, it could be applied to a broad pan-cancer mode to detect multiple cancer types simultaneously. It showed good performance in hepatocellular carcinoma (HCC) (75.0% sensitivity was achieved at 98.0% specificity) (22). Here we presented its utility in another cancer type, lymphoma. This study aimed to assess the potential for broad clinical utility of SeekInCare for lymphoma detection across diverse lymphoma subtypes.

## 2 Materials and methods

### 2.1 Sample collection

A total of 144 newly diagnosed and treatment-naïve lymphoma patients from two hospitals, The First Affiliated Hospital of Zhengzhou University and Sun Yat-sen University Cancer Center, were enrolled in this study from March 2021 to February 2022. Diagnosis and classification were made according to the 2016 revision of the World Health Organization classification of lymphoid neoplasms (5). 396 healthy individuals without any history of cancer or other cancer related clinical symptoms were enrolled as a control group. 99 healthy individuals from the physical examination center of the Second Affiliated Hospital of Sun Yat-sen University, with the remaining samples collected from our employees and their families. The study protocol was approved by the ethics committee of leading site: The First Affiliated Hospital of Zhengzhou University (2022-KY-0719-001). All participants provided written informed consent upon enrollment. One tube of blood (Streck, La Vista, USA) was collected from each participant after enrollment.

cfDNA extraction from blood, library construction, and sequencing process were described in a previous study by Meng et al, 2021 (22*).* In brief, cfDNA was extracted from plasma through the QIAamp Circulating Nucleic Acid Kit (Qiagen, Hilden, Germany). cfDNA was subject to library construction using the Kapa Hyper Prep Kit (Kapa Biosystems, Wilmington, MA) according to the manufacturer’s protocol. Prepared libraries were sequenced on NovaSeq system (Illumina, San Diego, CA), with pair-end 150 bp for WGS to generate approximately 10 Gb of raw sequencing data.

### 2.2 Protein tumor marker quantification

500 μL of plasma was used to quantify the expression of seven common protein tumor markers (AFP, CA125, CA15-3, CA19-9, CA72-4, CEA, and CYFRA21-1) by using Roche cobas e411 analyzer (Roche Diagnostics GmbH, Mannheim, Germany) and commercially available reagent kits following manufacturer’s instructions.

### 2.3 Sequence alignment and CNA analysis

The sequence alignment and CNA analysis were described in Meng et al, 2021 (22*).* In brief, human genome was divided into non-overlapping 1-Mb bins, using the reads number of each bin from healthy samples to calculate the mean value and standard deviation. Next, calculate the Z-score of each bin by subtracting the mean value and dividing by the standard deviation. Bins with an absolute Z-score greater than 3 were classified as CNA bins and samples with more than 10 such bins were deemed CNA positive.

In order to depict the significantly recurrent amplification/deletion regions in patients across different lymphoma subtypes, we divided the reads number of each bin by the mean value of corresponding bin, and performed log2-transformation (named log R ratio), which was used as input for QDNAseq (v1.22.0) package to detect CNA segments. Patients’ CNA segments were used to detect recurrent CNA amplification/deletion regions through GISTIC2.0 software (32).

### 2.4 FS analysis

Read pairs with a MAPQ score below 30 for either read or PCR duplicates were removed. Then calculate the length of each cfDNA fragment. The short fragments ratio (defined as P150) was the proportion of DNA fragments within 50~150bp. The ratio difference (named FS.S-L) between short and long (180~220bp) fragments was calculated as follows:


$$\text{FS}.\text{S}–\text{L}=\frac{\#\text{ of short reads }\left(50∼150\text{bp}\right)}{\#\;of\;total\;reads}\;-\;\frac{\#\;of\;long\;reads\;\left(180∼220bp\right)}{\#\;of\;total\;reads}$$

### 2.5 End motif analysis

The first 4-nucleotide sequence of each 5′ fragment end (Watson and Crick strands) was extracted from the mapping results and defined as end motif (16, 33, 34). Then Wilcoxon rank-sum test was used to detect the significantly different frequency of each end motif between lymphoma patients and healthy individuals, and use of false discovery rate (FDR) method to adjust for multiple testing. End motif with adjustment q-value less than 0.001 was selected to build a classifier to differentiate the lymphoma patients from the healthy subjects. To minimize the issue of overfitting and reduce the number of end motifs, least absolute shrinkage and selection operator regression (LASSO) method was applied to do the feature selection first. The selecting features were initially standardized, centering the data around a mean of zero and a standard deviation of one. This process was repeated 30 times and end motifs retained in all the repetitions were selected, which would be used to build the lymphoma predictive model by support vector machine (SVM). The average predicted value from multiple SVM models was used for further analysis.

### 2.6 EBV viral reads fraction calculation

Un-mapped reads were extracted from human mapping results. These reads were mapped to the Epstein–Barr virus (EBV) genome (AJ507799.2) with bowtie2 version 2.4.1 (http://bowtie-bio.sourceforge.net/index.shtml). After filtering the reads with mapping MAPQ score less than 30, the fraction of EBV reads per megabase was calculated as the following formula.


$$EBV\;viral\;read\;fraction=\frac{\#\;of\;reads\;mapped\;to\;EBV}{\#\;of\;total\;reads}\;\times \;10^{6}$$

### 2.7 Integrating multi-omics and multidimensional features to calculate cancer risk score

Through SeekInCare assay (**Supplementary Figure 1**), genomic features CNA, FS, end motif, and EBV value were analyzed by the sWGS data. Additionally, the protein tumor marker CA125 was incorporated as a feature. Feature selection was performed using LASSO followed by the construction of the lymphoma classifier model (35, 36). Before constructing the model, the five-dimensional features were initially normalized by subtracting the mean and dividing by the standard deviation.The sample set was randomly stratified in a 4:1 ratio into a training and validation set, then built the lymphoma classifier model with 10-fold cross-validation in the training set. This process was repeated 100 times. The average prediction value from these models was defined as CRS. If an individual’s CRS surpassed the average prediction value corresponding to a specificity of 98.0%, they would be categorized as having lymphoma

### 2.8 Statistical methods

The sample size for our study was determined using the Buderer’s method (37), aiming for a sensitivity of 75% at a specificity of 98% with a 95% confidence level and a case-to-control ratio of 1:2. The calculated values for n1 and n2 were 219 and 28, respectively, and the larger value was selected, resulting in a sample size of 219. Considering a 5% dropout rate, the final sample size was set at 231. Notably, we were able to surpass this requirement by collecting a total of 540 samples for our study. This larger sample size not only meets the study’s statistical power needs but also contributes to heightened result reliability.

All statistical analyses were performed using R statistical software (version 4.1.2), which was described in a previous study by Meng et al, 2021 (22).

## 3 Results

### 3.1 Characteristics of enrolled participants

We prospectively enrolled 144 treatment-naïve lymphoma patients and 396 healthy individuals in this study. The mean ages were 51.2 years in the lymphoma group and 46.9 years in the healthy group (p< 0.01, **Table 1**). Sub-classification of lymphoma patients was summarized in **Table 1** (**Table 1**). There was no significant difference between the two groups in gender (p = 0.144). As for lymphoma classification, except for 14 HL patients, 130 (90.3%) lymphoma patients were non-Hodgkin lymphoma (NHL), 76.2% of which originated from B cells and 23.8% from T/NK cells. Among B-cell NHLs, 79.8% were aggressive types, including diffuse large B-cell lymphoma (DLBCL), accounting for 82.3% of the total subgroup. The detailed information of each sample was shown in **Supplementary Table 1**.

**Table 1:** Demographics and clinical information of lymphoma patients and healthy individuals.

	Lymphoma	Healthy	p value*
	(n = 144)	(n = 396)	
Age(mean ± SD)	51.2 ± 16.4	46.9 ± 13.1	0.005
<45	49 (34.0%)	160 (40.4%)	<0.001
45~55	26 (18.1%)	118 (29.8%)	
55~65	37 (25.7%)	78 (19.7%)	
>65	32 (22.2%)	40 (10.1%)	
Gender			
F	67 (46.5%)	214 (54.0%)	0.144
M	77 (53.5%)	182 (46.0%)	
Stage			
I	19 (13.2%)		
II	20 (13.9%)		
III	15 (10.4%)		
IV	65 (45.1%)		
*NA*	*25 (17.4%)*		
Major subtype			
HL	14 (9.7%)		
NHL	130 (90.3%)		
NHL Cell Origin			
T/NK-cell	31 (23.8%)		
B-cell	99 (76.2%)		
B-cell NHL aggressive or not			
No (*Indolent*)	20 (20.2%)		
Yes (Aggressive)	79 (79.8%)		
DLBCL or not in aggressive B-cell NHL group			
No	14 (17.7%)		
Yes	65 (82.3%)		

Values are n (%).

*Student’s t-test was used to compare mean age difference between healthy and lymphoma, and Fisher’s exact test was used to estimate the difference of age and gender compositions between those two groups; SD, standard deviation; HL, Hodgkin lymphoma; NHL Non-Hodgkin lymphoma.

### 3.2 Protein tumor markers for lymphoma diagnosis

**Figure 1:**
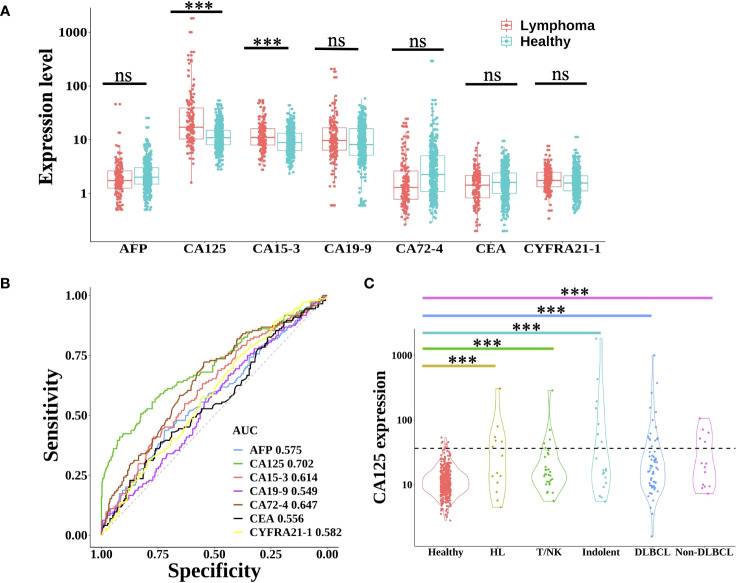
Expression level of protein tumor markers between lymphoma and healthy group. **(A)** Boxplot plots of seven protein tumor markers (AFP, CA125, CA15-3, CA19-9, CA72-4, CEA, and CYFRA21-1) expression in lymphoma patients and healthy individuals. Statistical significance of the increasing trend of lymphoma patients was evaluated by the Wilcoxon rank-sum test. **(B)** The receiver operating characteristic (ROC) curve of each protein tumor marker showed the performance for lymphoma prediction. **(C)** Violin plots showed the expression of CA125 in healthy individuals and different lymphoma subtypes. The dotted line indicated the CA125 clinical cut-off value of 35 U/ml. HL, Hodgkin lymphoma; T/NK, T/NK cell lymphoma; Indolent, indolent B-cell lymphoma; DLBCL, diffuse large B-cell lymphoma; ns, not significant; ***, p< 0.001 (Wilcoxon rank-sum test).

Protein tumor markers have been used for decades to aid in the diagnosis and management of a variety of cancers. Here, seven common plasma protein tumor markers (AFP, CA125, CA15-3, CA19-9, CA72-4, CEA, and CYFRA21-1) were selected to estimate the expression in each lymphoma patient and healthy individual (18). As shown in **Figure 1A**, only the expression of CA125 and CA153 in lymphoma was significantly higher than that in the healthy group (Wilcoxon rank-sum test, P<0.001). However, using receiver operating characteristic (ROC) analysis to evaluate these proteins’ performance in differentiating lymphoma from healthy subjects (**Figure 1B**), only CA125 achieved a moderate AUC (area under the curve) of 0.702. Based on the clinical cut-off value: 35 U/ml, CA125 positive ratio was only 2.3% in healthy individuals and 27.8% in lymphoma patients (24.7% in male and 31.3% in female lymphoma respectively) (**Supplementary Table 2**), which indicated CA125 could be used as a biomarker for lymphoma detection, regardless of gender. As for the different subtypes of lymphoma, CA125 positive ratio was 42.9% in HL. In NHL, CA125 positive proportion of indolent B-cell lymphoma was 40.0%, which was higher than aggressive B-cell lymphoma (26.6%). The positive ratio for T/NK cell lymphoma was the least at 16.1% (**Figure 1C**).

### 3.3 Distinct CNA patterns across diverse lymphoma subtypes

Since somatic CNA has been widely detected in most cancer patients, sWGS was used to depict the CNA profile of lymphoma patients and healthy subjects. 88 (61.1%) patients were CNA positive, however only 5 (1.3%) healthy subjects were CNA positive, which suggested that CNA might be an efficient feature for lymphoma diagnosis with high specificity of 98.7% (**Supplementary Figure 2A**). Especially for HL, 85.7% of patients were CNA positive. Aggressive B-cell NHL patients had the second highest CNA mutational frequency. Among them, DLBCL and Non-DLBCL had similar CNA mutational frequency (64.6% vs 71.4%). The CNA positive ratio was lowest in T/NK-cell NHL (41.9%).

**Figure 2:**
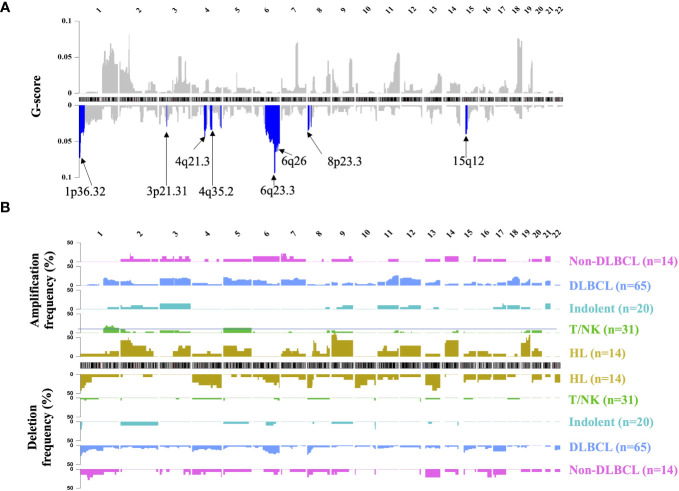
Landscape of genomic CNA detection in ctDNA from lymphoma patients. Chromosomal overview of focal recurrent amplification (upper) and deletion (lower) regions was shown in **(A)**, and the G-score was calculated by the GISTIC analysis. The blue region represented the significant focal deletion region (q-value = 0.25) with the annotated position. Fractions of aberrant samples across genomic loci and different lymphoma subtypes were shown in panel **(B)** HL, Hodgkin lymphoma; T/NK, T/NK cell lymphoma; Indolent, indolent B-cell lymphoma; DLBCL, diffuse large B-cell lymphoma.

Segmented CNA profiles of each lymphoma patient was shown in **Supplementary Figure 2B**. The heatmap showed that aggressive B-cell lymphoma (including DLBCL and non-DLBCL) had a significantly higher absolute value of log R ratio of CNA segments than the other subtypes, which meant aggressive B-cell patients had a higher concentration of ctDNA in plasma.GISTIC analysis was used to find the significant recurrent CNA peaks in lymphoma and the genomic region mutational frequency across different lymphoma subtypes. In all the patients, significant mutational focal deletion peaks (1p36.32, 3p21.31, 4q21.3, 4q35.2, 6q23.3, 6q26, 8p23.3, and 15q12) and broad amplification regions (1q, 3p, 3q, 5p, 5q, 7p, 7q, 9p, 12p, 12q, 18q, 19q, and 21q) were identified (**Figure 2A**, **Supplementary Table 3**). The CNA mutational pattern of each lymphoma subtype with depicted in **Figure 2B**. Among HL patients, chromosomal gains most frequently involved 2p, 9, 14, and 19, whereas losses primarily affected chromosomes 1p, 4q, and 13q. Amplification of 1q, 3, 7, 11q, 12, 18q and deletion of 1p, 6q, 8p, and 15p was frequently mutated in the DLBCL, which was similar to the Non-DLBCL CNA mutational frequency. T/NK-cell NHL did not have frequent mutational regions, except for amplification of 1q and 5. CNA pattern differences among these major subtypes might contribute to identifying the lymphoma subtypes.

### 3.4 Characterizing aberrant fragment size distribution of cfDNA in lymphoma patients

**Figure 3:**
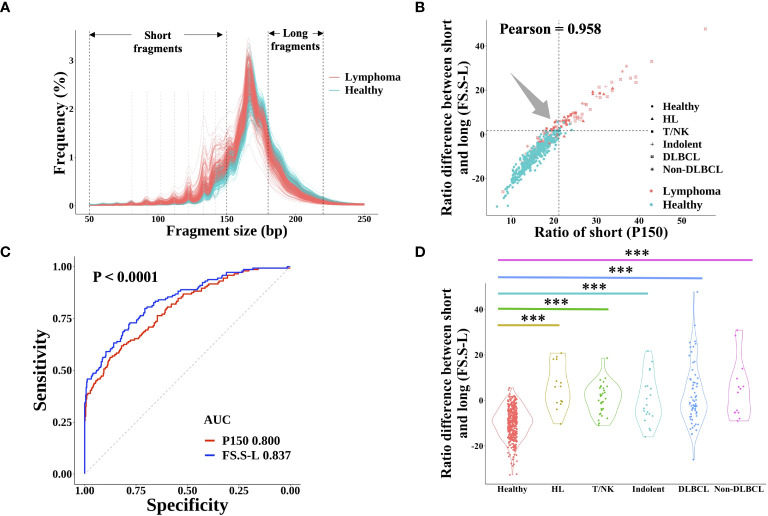
cfDNA fragment size distribution differentiated between lymphoma patients and healthy subjects. **(A)** The fragment size distribution of cfDNA was determined by sWGS of plasma samples from lymphoma patients and healthy subjects. The black dotted line showed the range of short fragments (50~150bp) and long fragments (180bp~220bp). The grey dotted line showed the location of 10-periodic peaks (81bp, 92bp, 102bp, 112bp, 122bp, 133bp, and 142bp). The correlation between the proportion of short fragments (defined P150) and the proportion difference between short and long fragments (defined FS.S-L) was shown in **(B)** The dotted line represented the cut-off value at 98% quantiles in the healthy group. **(C)** ROC curve of P150 and FS.S-L showed the performance for lymphoma prediction. DeLong’s test was used to show that the AUC of FS.S-L (blue) was significantly better than the AUC of P150 (red) with p< 0.0001. **(D)** Compare the value of FS.S-L between healthy individuals and different lymphoma subtypes. Statistical significance of the increasing trend of P150 for each lymphoma subtype was determined by the Wilcoxon rank-sum test (***p< 0.001). HL, Hodgkin lymphoma; T/NK, T/NK cell lymphoma; Indolent, indolent B-cell lymphoma; DLBCL, diffuse large B-cell lymphoma.

It is well known that cfDNA from cancer patients had significantly more short fragments than that from healthy individuals, which could be used for cancer prediction (21). However, there are few published studies about the FS profile in lymphoma. In this study, the fragment size distribution in both lymphoma and healthy individuals was shown in **Figure 3A**. Similar to the FS distribution of solid tumors, lymphoma patients had a higher proportion of short fragments (50~150 bp, P150) than healthy subjects and the same profile of 10-periodic peaks (21, 22). Meanwhile, the proportion of long fragments (180~220 bp) in lymphoma patients was lower than that in healthy control. Based on these results, we developed a new index of FS (named FS.S-L, Materials and Methods), which had a high correlation with the previous FS method P150 (Pearson: 0.985) and had a better performance for cancer prediction. At the same specificity of 98.0%, FS.S-L predicted 6.9% more lymphoma patients than the P150 (**Figure 3B**), and FS.S-L index (AUC = 0.837) also has a significantly better performance (p< 0.0001 by DeLong’s test) to discriminate lymphoma and healthy individual than P150 (AUC = 0.800) (**Figure 3C**). The value of FS.S-L in each lymphoma subtype was also significantly higher than that in healthy individuals by the Wilcoxon rank-sum test (p< 0.001) (**Figure 3D**), which suggested that FS.S-L was suitable for all the lymphoma detection, regardless of subtypes.

### 3.5 Differential end motif proportions between lymphoma patients and healthy individuals

**Figure 4:**
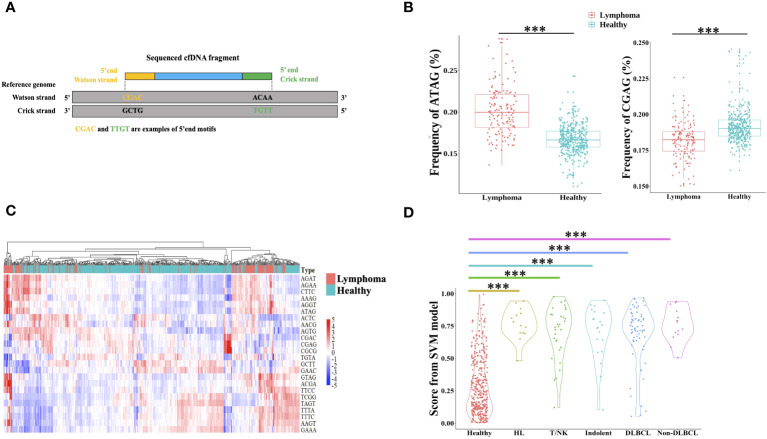
Differential end motif profiles between lymphoma patients and healthy individuals. **(A)** illustrated the determination of plasma DNA end motifs. After aligning the pair-end reads to the reference genome, the first 4-nucleotide sequence on each 5′ fragment end (Watson and Crick strands) of one fragment DNA was defined as end motif. As shown in the example, CGAC and TTGT was the example of end motifs. One sample included a total of 256 4-mer motifs. **(B)** Boxplot showed two examples of the frequency difference of selected end motifs between lymphoma subjects and healthy individuals. **(C)** Heatmap analyzed the frequencies of 24 selected end motifs between healthy and lymphoma subjects, and samples were clustered based on the frequency of selected end motifs. The data are row normalized. **(D)** Violin plot showed the lymphoma predicted score by support vector machine. HL, Hodgkin lymphoma; T/NK, T/NK cell lymphoma; Indolent, indolent B-cell lymphoma; DLBCL, diffuse large B-cell lymphoma. ***, Wilcoxon rank-sum test P-value< 0.001.

The cfDNA end motif was identified using the first 4bp sequence on each 5′ end of cfDNA fragment after alignment to the reference genome (**Figure 4A**). 189 out of 256 end motifs with a different frequency between lymphoma patients and healthy individuals were found by the Wilcoxon rank-sum test and p-value adjust method (FDR). For example, the frequency of motif ATAG showed a significant increase in lymphoma patients, while the frequency of motif CGAG was significantly lower in lymphoma patients (**Figure 4B**, Materials and Methods). To minimize the issue of overfitting, LASSO was used to select the end motifs and reduce the number of features. Finally, 24 end motifs were retained in the feature selection process by LASSO after repeating 30 times. Hierarchical clustering analysis was used to identify the different characteristics of selected end motifs between the cancer patients and healthy controls (**Figure 4C**). Heatmap formed different clusters between lymphoma patients and healthy subjects and showed that the frequency of selected end motifs in lymphoma patients was quite distinct from that in healthy individuals. Use the frequency of selected end motifs to build the lymphoma predictive model by SVM, and the predicted score of healthy subjects and lymphoma patients across different subtypes was shown in **Figure 4D**. All the lymphoma subtypes had a significantly higher predicted score than healthy controls.

### 3.6 Quantitative detection of EBV by the sWGS

In addition to the lymphoma specific ctDNA genomic feature, quantification of circulating EBV DNA was useful for diagnosis, monitoring, and prognostication of lymphoma, especially for HL and T/NK-cell NHL patients (38–40). Based on the sWGS data, the proportions of circulating EBV reads in lymphoma patients and healthy individuals were shown in **Supplementary Figure 3**. 42.9% HLs and 54.8% T/NK-cell NHLs were EBV positive, with more than 0.5 reads per million. However, the plasma EBV proportions of all healthy individuals were far less than 0.5 reads/M and the maximum was 0.16 reads/M. EBV status of other lymphoma subtypes (Indolent, DLBCL, and non-DLBCL) was similar to that of healthy subjects. 21 lymphoma patients also accepted EBV detection by real-time PCR in blood samples and *in situ* hybridization in tissue samples. 5 patients with EBV negative in tissue were also negative in blood, regardless of method. 15 out of 16 patients (93.8%) with EBV positive in tissue were also EBV positive detected by sWGS in blood, but only 5 patients (31.3%) were detected by the real-time PCR method in the blood (**Supplementary Table 4**). These findings suggested that our assay was more sensitive for EBV detection in blood than the real-time PCR method, which could be used for lymphoma detection.

### 3.7 The blood-based multidimensional assay achieved optimal accuracy in lymphoma detection

**Figure 5:**
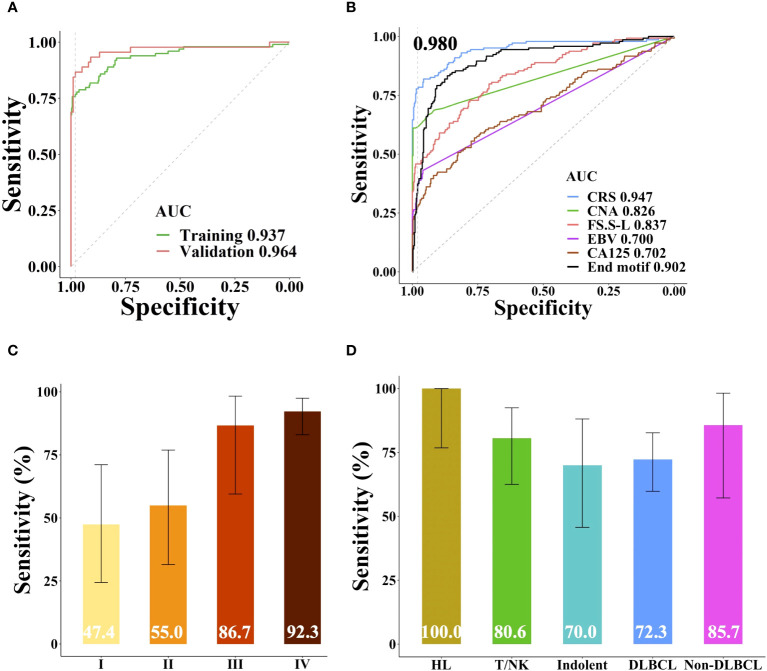
Performance of SeekInCare assay in lymphoma prediction. **(A)** Performance comparison of SeekInCare assay in the training cohort and validation cohort. (Delong’s test p = 0.3). **(B)** ROC analysis comparing different dimensions in classification of lymphoma patients (n = 144) from healthy individuals (n = 396) by CA125, CNA, FS, EBV, end motif, and the integrating value named CRS. Sensitivities at 98.0% specificity across different tumor stages and lymphoma subtypes were shown in **(C, D)** respectively. The error bar indicated the range of 95% confidence interval.

Based on the sample source, samples were divided into training cohort and validation cohort. 45 patients and 99 healthy individuals were enrolled from different affiliated hospitals of Sun Yat-sen University were used as independent validation cohort, the remaining samples were used as training cohort to build the lymphoma classifier model, which achieved AUC of 0.937. SeekInCare performance in the validation cohort mirrored that of the training cohort, with an AUC of 0.964 (Delong’s test p = 0.3, **Figure 5A**), affirming its generalizability. Therefore, we amalgamated all samples for further assessment of each feature’s independent performance in distinguishing lymphoma patients from healthy individuals via ROC analysis and calculated the CRS value. CNA made the largest contribution to the model, which was the highest sensitivity of all the five variables at specificity of 98.0% followed by the FS, end motif, EBV and CA125 (**Figure 5B**). Pearson correlation among these five features was shown in **Supplementary Figure 4A**. Apart from a correlation of 0.57 between CNA and FS.S-L, and a correlation of 0.55 between FS.S-L and the end motif, there was no correlation among the other features, CA125, end motif, and EBV. SeekInCare detected 112 out of 144 lymphoma patients [77.8% (95% CI, 70.1%~84.3%)] at 98.0% specificity (95% CI, 96.1%~99.1%) (**Supplementary Table 5**), and the overall accuracy is 92.6% (95% CI, 90.0%~94.7%). Both the positive predictive value (PPV) and negative predictive value (NPV) were more than 92%. Meanwhile, the AUC of CRS was 0.947 (**Figure 5B**) and the AUC of precision-recall curve was 0.920 (**Supplementary Figure 4B**). The sensitivity of lymphoma detection was increased with the advancement of stages. While the sensitivity of early stage was 51.3% (47.4% (95% CI, 24.4%~71.1%) and 55.0% (95% CI, 31.5%~76.9%) for stage I and II respectively), and the sensitivity of advanced stage was 90.0% (stage III: 86.7% (95% CI, 59.5%~98.3%); Stage IV: 92.3% (95% CI, 83.0%~97.5%)), with high specificity of 98.0% (**Figure 5C**). Among lymphoma subtypes, all the HL patients were successfully detected, including 33.3% of early stage HLs. For the aggressive B cell lymphoma, including both DLBCL and non-DLBCL with a sensitivity of 72.3% (95% CI, 59.8%~82.7%) and 85.7% (95% CI, 57.2%~98.2%), respectively (**Figure 5D**). These results suggested that SeekInCare was an efficient non-invasive assay for lymphoma detection with high accuracy.

### 3.8 Dynamic changes of blood-based assay correlate with treatment outcome

**Figure 6:**
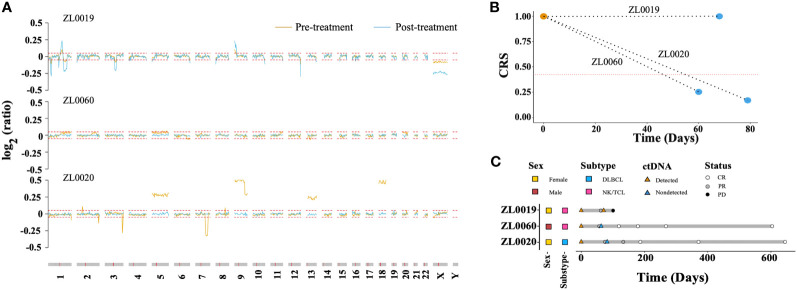
Molecular response assessment of serial plasma samples. **(A)** Dynamics of the CNA profile before treatment (brown) and after 2 treatment cycles (baby blue). The red dotted line represented the cutoff value of the log R ratio for CNA ( ± 0.05). The CRS values before treatment and after 2 treatment cycles are shown in **(B)** The red dotted line represents the cut-off value of CRS at 98% specificity. A CRS value exceeding this cutoff is defined as ‘ctDNA detected,’ while a value below is categorized as ‘ctDNA not detected’. Molecular and imaging responses are illustrated in **(C)**.

We explored the CRS dynamic changes after lymphoma related treatment. Blood samples were collected from three patients with stage IV lymphoma prior to treatment and after completing two treatment cycles. Notably, the amplitude of CNAs in patient ZL0019, exhibited a significant increase in regions such as 1q, 9p, and X (**Figure 6A**). In contrast, CNAs that were initially detected in the samples from patients ZL0020 and ZL0060 prior to treatment had disappeared, with no further detection of CNAs in their plasma after treatment. Concurrently, the other dimensions, including FS.S-L, CA125, end motif, and EBV, exhibited the changes consistent with the observed changes of CNAs. Specifically, The value of these dimensions increased in ZL0019 post-treatment, while they decreased to normal levels in ZL0020 and ZL0060, except EBV in ZL0020 and FS.S-L in ZL0019 remained negative (**Supplementary Figure 5**). Furthermore, our analysis revealed that CRS of ZL0019, as predicted by GLM, approached more closely to 1 after treatment (from its pre-treatment value of 0.9979 to 0.9995). Although several individual dimensions exhibited a noteworthy increase in ZL0019, the nature of the sigmoid curve within the GLM model posed a challenge in precisely quantifying this change (**Figure 6B**). Additionally, the CRS values for ZL0020 and ZL0060 decreased to the normal level.

Remarkably, after two treatment cycles, all three patients exhibited a Partial Response (PR) according to image evaluations. However, after four cycles, ZL0019 progressed to Progressive Disease (PD), while ZL0060 achieved a Complete Response (CR), and ZL0020 also reached CR after six treatment cycles (**Figure 6C**). These findings illustrated the potential of liquid biopsy in promptly assessing the effectiveness of cancer treatment, as they align with clinical evaluations following two treatment cycles.

## 4 Discussion

Lymphomas, encompassing diverse neoplasms arising from B lymphocytes, T lymphocytes, and NK cells, are characteristic tumors surrounded by an inflammatory microenvironment. Among them, DLBCL constitutes a major portion of NHLs, displaying high clinical and biological heterogeneity because it arises from germinal center B cells at different stages of differentiation (41). Its classification is intricate, constantly evolving due to heterogenic variations in morphology, phenotype, genetic anomalies, prognosis, and clinical features (42). The tumor microenvironment (TME) is a complex composed environment enveloping cancer cells, comprising both cellular and extracellular elements, as well as a vascular network (43, 44). TME components play pivotal roles in both initiating and sustaining carcinogenesis (45). The composition of TME cells holds relevance for the prognosis of B-cell lymphomas, including cHL, DLBCL and FL (46). It plays a pivotal role in various biological processes such as pathogenesis, progression, metastasis and drug resistance, facilitating sustained proliferation and immune escape (47, 48). Tumor progression is profoundly shaped by the TME. Via various pathways, tumor cells adeptly recruit stromal cells, providing tumor cell growth signals, intermediate metabolites, and a conducive environment for tumor progression and metastasis (49). Angiogenic factors, like vascular endothelial growth factor and its receptors, along with other TME components, are crucial in the progression and maintenance of lymphoproliferative disorders (50). Increased interfollicular microvascular density in FL patients predicts inferior overall survival and an increased transformation to DLBCL (51, 52). Antiangiogenic therapy has emerged as a vital tool for lymphoma treatment (53). In the bone marrow microenvironment, interactions with stromal cells and the extracellular matrix contribute to cell adhesion-mediated drug resistance, impacting chemotherapy efficiency and prognosis in B-NHL (54). Knockdown of small glutamine-rich TPR-containing protein A (SGTA) in Non-Hodgkin’s Lymphomas induced CAM-DR, offering the potential for a novel therapeutic approach for CAM-DR in NHL (55).

Lymphoma is often diagnosed at an advanced stage when it has caused symptoms, and the diagnosis and classification are mainly based on the excisional or punctured tissues. It has been challenging to develop a more sensitive and specific method of detecting lymphoma when it was still asymptomatic. With the advance of ‘liquid biopsy’ and next-generation sequencing technology, we developed a blood-based non-invasive assay, SeekInCare, which integrated the protein marker and multidimensional cancer genomic features, such as CNA, FS, end motif, and oncogenic viral DNA. In this study, the SeekInCare assay has achieved an impressive 77.8% sensitivity with the high specificity of 98.0%, and 92.6% accuracy, while its NPV and PPV were both more than 92% for lymphoma detection. The assay also performed well in detection of early stage lymphoma as the sensitivity of early stage was 51.3% (47.4% and 55.0% for stage I and II respectively).

CA125 is used to monitor certain cancers during and after treatment, especially ovarian cancer in symptomatic women presenting to primary care (56). Interestingly, among 7 commonly used plasma protein tumor markers for cancer, we found that only CA125 could be effectively used for lymphoma detection. It is especially effective in HL with a sensitivity of 42.9% and high specificity of 98.0%, compared to a moderate sensitivity of 27.8% at the same specificity for all the lymphoma patients in this study. Moreover, CA125 for lymphoma detection had the same performance between female and male patients, suggesting that CA125 is not female-specific as we may think in ovarian cancer. Therefore, our study suggests that CA125 can be a potential biomarker in lymphoma, especially in HL, for diagnosis.

The CNA analysis demonstrated that aggressive B-cell patients had a higher concentration of ctDNA in plasma which might be the reason for aggressive B-cell lymphoma patients who usually had a poor prognosis (57). We also found CNA from our assay could also reveal some lymphoma critical genes, such as *TNFRSF14* in 1p36.32, *TNFAIP3* in 6q23.3, and *SETD2* in 3p21.31. These cancer genes play an important role in immune escape and apoptotic response, which might benefit the strategy for lymphoma therapy and predict the prognosis. Specifically, mutations and deletions in the *TNFRSF14* gene are common in follicular lymphoma (FL), increasing the ability of lymphoma cells to stimulate allogeneic T-cell responses. FL patients with *TNFRSF14* aberrations may benefit from more aggressive immunosuppression to reduce harmful graft-versus-host disease after transplantation (58). Loss of *TNFAIP3* enhances MYD88_L265P_-driven signaling in non-Hodgkin lymphoma, presenting a potential opportunity for therapeutic targeting (59). Each subtype had a different CNA positive ratio and some unique frequently amplified/deleted regions, such as HL are frequently amplified at 2p, 9, 14, 19 and deleted at 1p, 4q, 13q. These genomic regions were also reported to be frequently affected by CNA in previous HL studies (60, 61). In addition to lymphoma diagnosis, the CNA profiles could be used to identify lymphoma subtypes and predict prognosis to some extent.

Similar fragmentation patterns were found in lymphoma as in solid tumors, such as HCC, showing the 10-bp periodic peaks located between 80 and 150 bp size ranges as previously reported (22). We have tried several methods to estimate the difference in FS between lymphoma patients and healthy individuals. We found a method to calculate the FS.S-L, which had a better performance than the previously published method P150.

As cleavage and fragmentation of cfDNA are nonrandom processes, cleavage site preference can be associated with tissue sources, disease status, chromatin accessibility, and nuclease activities (16, 33, 62). Thus, we were the first to depict the fragment-omics character of lymphoma based on end motifs and found a specific cluster of end motif frequency. Just like the HCC study (63), we also demonstrated that the end motif could be used to differentiate lymphoma from healthy subjects, which achieved an AUC of 0.902.

Quantitative detection of EBV by sWGS in blood achieved high sensitivity (93.8%) at 100% specificity, which was much more sensitive than real-time PCR (31.3% sensitivity). One fact that people with EBV positive might be caused by other diseases (e.g., ongoing infectious mononucleosis) irrespectively of lymphoma. Nonetheless, the combination of EBV quantification and typical lymphoma alterations of other dimensions (CNA/end motif) strongly indicates an EBV-driven lymphoma. Meanwhile, EBV was also used as a molecular biomarker for disease monitoring and prognosis prediction in EBV positive patients, since pathogenesis and genetics were different between EBV-positive patients and EBV-negative patients, especially in HL, Burkitt lymphoma and some subtypes of T/NK-cell NHL (38, 39, 64). Even more, the SeekInCare assay was not only more sensitive to monitoring EBV expression in the blood than the real-time PCR method, but also reflected the dynamic change of lymphoma-associated mutations (CNA).

While the blood samples were collected from only three patients after two treatment cycles, this nevertheless underscores the potential of plasma-based molecular dynamics in promptly and accurately assessing treatment effectiveness. This finding is consistent with previous studies that demonstrated ctDNA levels after two treatment cycles serving as prognostic factors, with ctDNA dynamics significantly associated with clinical outcomes in DLBCLs (57, 65). This capability is particularly crucial in distinguishing genuine treatment responses from pseudo-responses (66).

This study had some limitations. More prospectively collected samples from multiple centers or real-world scenarios are needed to validate the generalizability of SeekInCare. Even though the assay demonstrated with high specificity of 98.0%, there are still 2.0% positive that require those individuals to go through routine clinical workup for confirmation including CT scan and/or biopsy. SeekInCare for lymphoma detection utilized common cancer hallmarks, not specific characteristics of lymphoma (such as AFP for HCC). We found the blood markers associated with lymphoma tumor burden including lactate dehydrogenase (LDH) and beta 2 microglobulin (β2M) were also informative with 38.6% positivity for LDH and 30.8% for β2M based on the clinical cut-offs in the current study. We could build a baseline in a healthy cohort to optimize the specificity of LDH and β2M combining with the features of SeekInCare for lymphoma early detection in a future study.

Recent studies have demonstrated that fragment-omics features from sWGS could be used to predict tissue origin of cancer (21, 67), and it would be optimized in future studies. Therefore, lymphoma would be differentiated from other cancers through the TOO algorithm and lymphoma patients could benefit from blood-based non-invasive early detection. Indeed we were able to identify nine lymphoma patients out of total 41 cancer cancer cases in our proof-of-concept study utilizing CNAs and the seven protein markers (18). SeekInCare could be used as a complementary test to medical imaging to distinguish the benign and malignant lymph nodes, especially for patients with that affected nodes were difficult to reach, or tissue-based subtyping was inconclusive. In summary, a non-invasive assay (SeekInCare) was validated in our case-control study, achieving an AUC of 0.947 for lymphoma detection, which also provided insights into lymphoma monitoring and multi-cancer early detection.

## Data availability statement

The raw data for both healthy individuals and lymphoma patients are accessible with controlled access through the Chinese National Genomics Data Center under PRJCA022396 (https://ngdc.cncb.ac.cn/bioproject/browse/PRJCA022396).

## Ethics statement

The studies involving humans were approved by The First Affiliated Hospital of Zhengzhou University. The studies were conducted in accordance with the local legislation and institutional requirements. The participants provided their written informed consent to participate in this study.

## Author contributions

YC: Data curation, Resources, Writing – review & editing. SL: Formal Analysis, Writing – original draft. ZL: Data curation, Resources, Writing – review & editing. XW: Resources, Supervision, Writing – review & editing. FC: Project administration, Writing – review & editing. SG: Formal Analysis, Writing – review & editing. DZ: Formal Analysis, Methodology, Writing – review & editing. GZ: Writing – review & editing. WW: Formal Analysis, Writing – review & editing. YYC: Investigation, Project administration, Writing – review & editing. ST: Writing – review & editing. MM: Funding acquisition, Methodology, Supervision, Writing – review & editing.

## Supplementary material

Supplementary Figure 1 Schematic of multi-omics and multidimensional SeekInCare approach. One tube (8ml) of peripheral blood was collected from healthy individuals and lymphoma patients. cfDNA was extracted and then analyzed the cancer genomics, epigenetics, and virus infection through shallow whole genome sequencing. Meanwhile, the expression of seven protein tumor markers was also measured in the plasma. Cancer risk score (CRS) was calculated by machine learning method, which integrated CNA, FS, end motif, virus, and protein marker. CNA, copy number aberration, WGS, whole genome sequencing.

Supplementary Figure 2 **(A)** Violin plot showed the number of CNA bins in healthy individuals and patients across different lymphoma subtypes. Since the y-axis was a logarithmic scale, sample without CNA bins was changed to 0.1. The dotted line indicated the cut-off value of CNA positive. **(B)** The Heatmap showed the segmented CNA profiles of all lymphoma patients. The chromosomes were arranged vertically from top to bottom, and lymphoma samples were sorted by the genomic region size of CNA among each lymphoma subtype from left to right. Red and blue represent amplification and deletion, respectively. HL, Hodgkin lymphoma; T/NK, T/NK cell lymphoma; Indolent, indolent B-cell lymphoma; DLBCL, diffuse large B-cell lymphoma.

Supplementary Figure 3 The proportion of plasma EBV DNA reads among the total number of sequenced plasma DNA reads in healthy individuals and lymphoma patients. The cut-off value was 0.5 reads/M (dotted line). HL, Hodgkin lymphoma; T/NK, T/NK cell lymphoma; Indolent, indolent B-cell lymphoma; DLBCL, diffuse large B-cell lymphoma.

Supplementary Figure 4 **(A)**The Pearson correlation between different features among all samples. **(B)** Precision-recall curve for the CRS values obtained from the SeekInCare assay in the classification of lymphoma patients. CNA, copy-number aberration. FS.S-L, the ratio difference between short and long fragments. EBV, Epstein–Barr virus.

Supplementary Figure 5 The dynamic changes in each feature before and after treatment were illustrated. The dotted line represented the cut-off value set at the 98th percentile within the healthy group.
