# Supplementary figures and images for: Non-invasive detection of lymphoma with circulating tumor DNA features and protein tumor markers
Source: Front Oncol. 2024 Jan 19;14:1341997. doi: 10.3389/fonc.2024.1341997 (PMC10834776; doi:10.3389/fonc.2024.1341997)

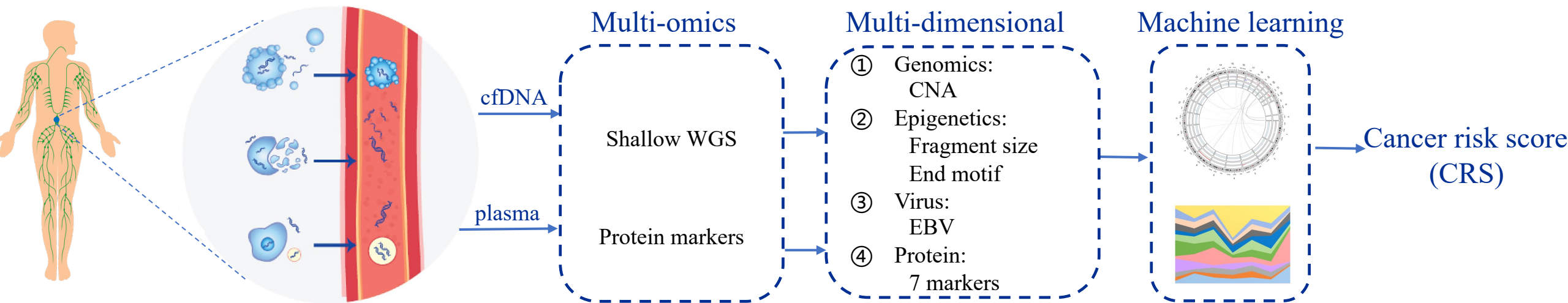

**A**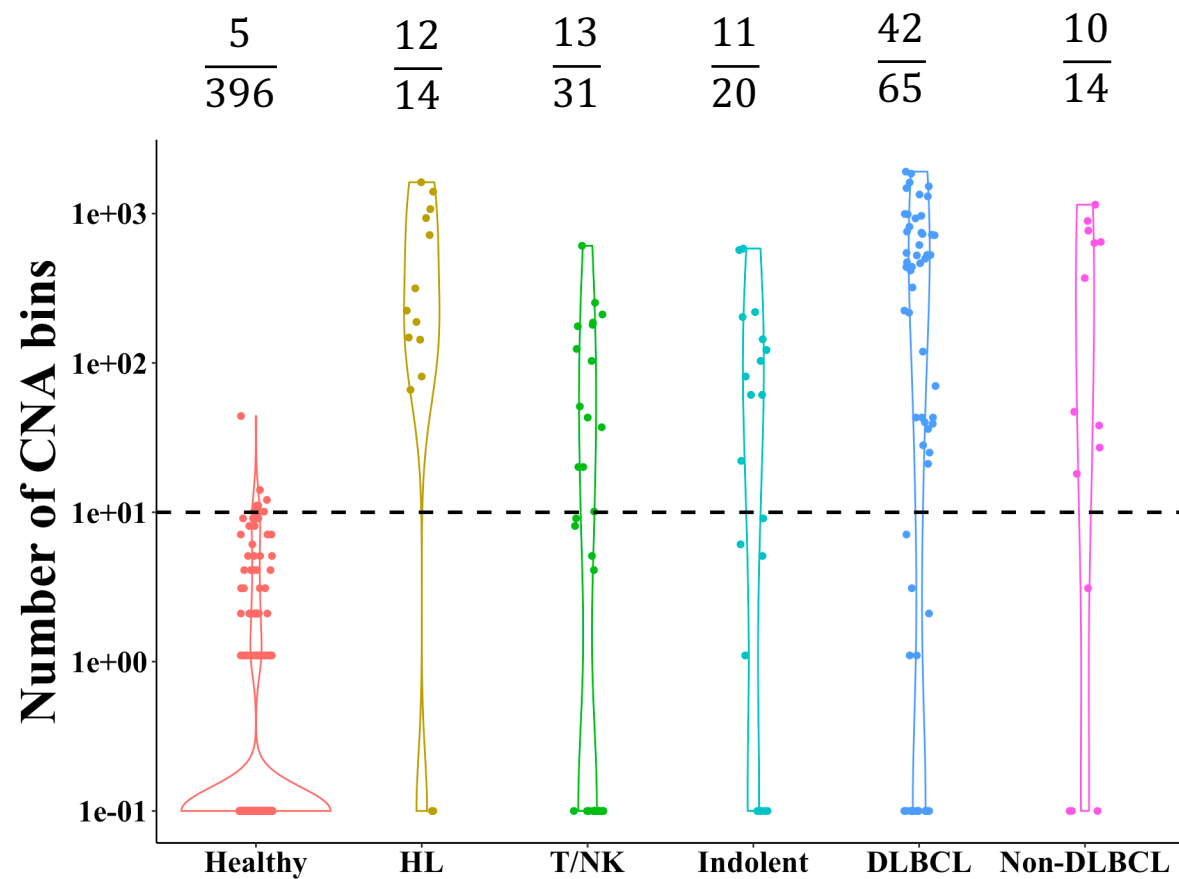**B**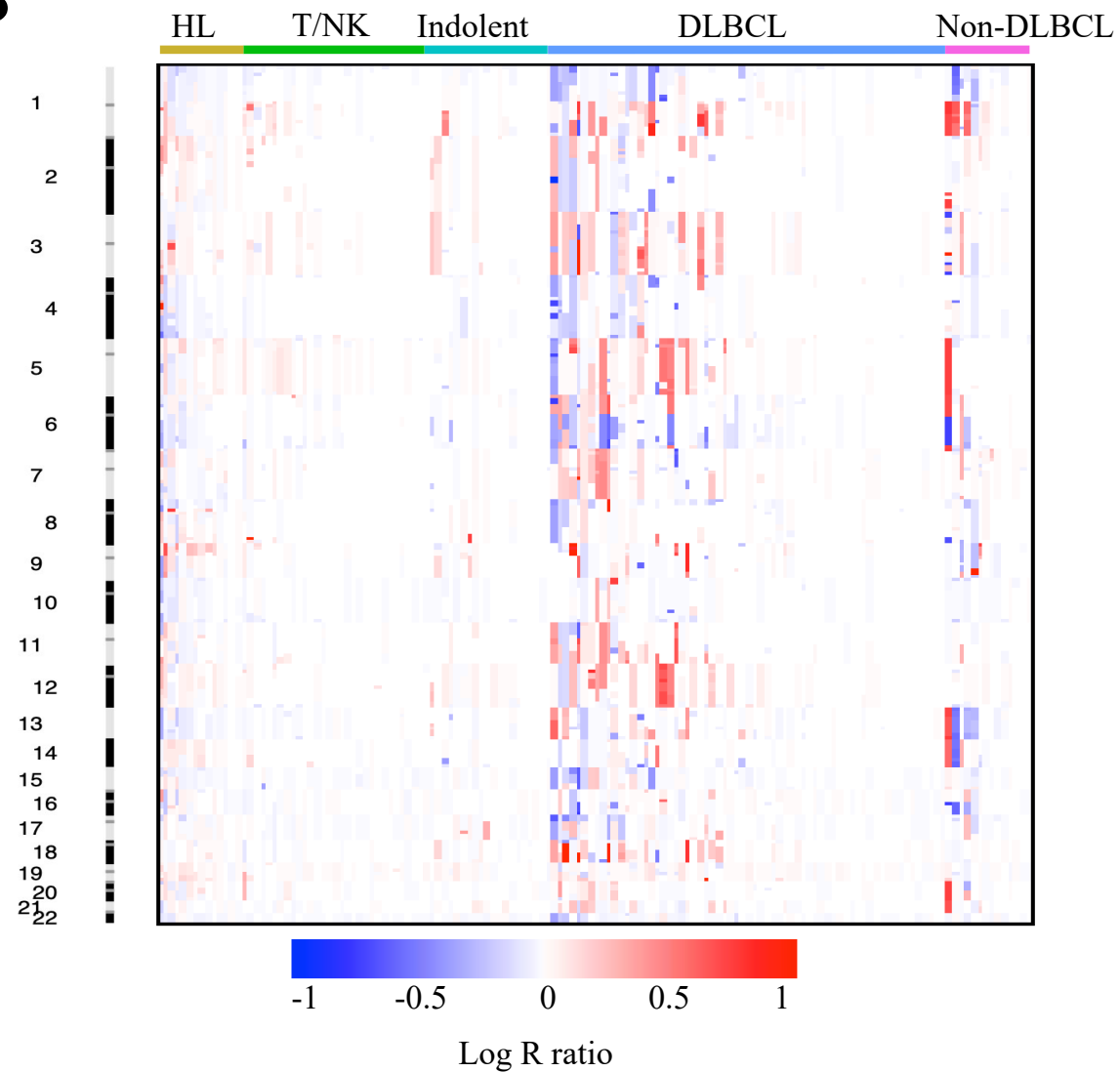

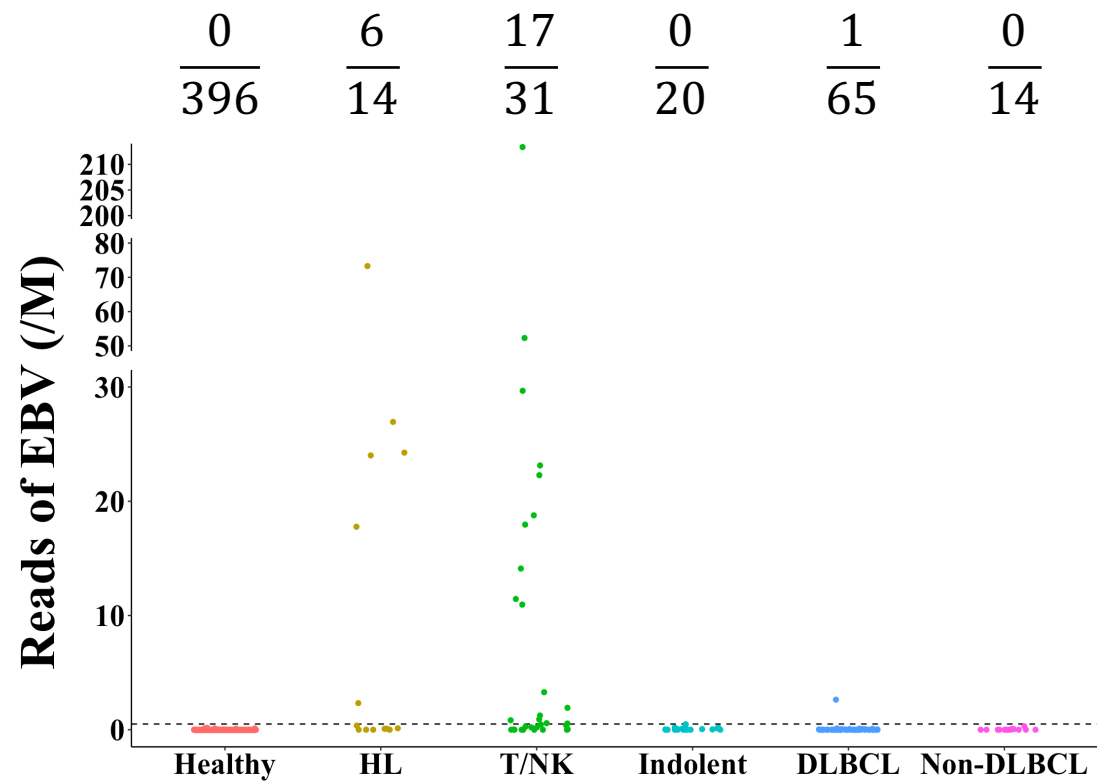

**A**

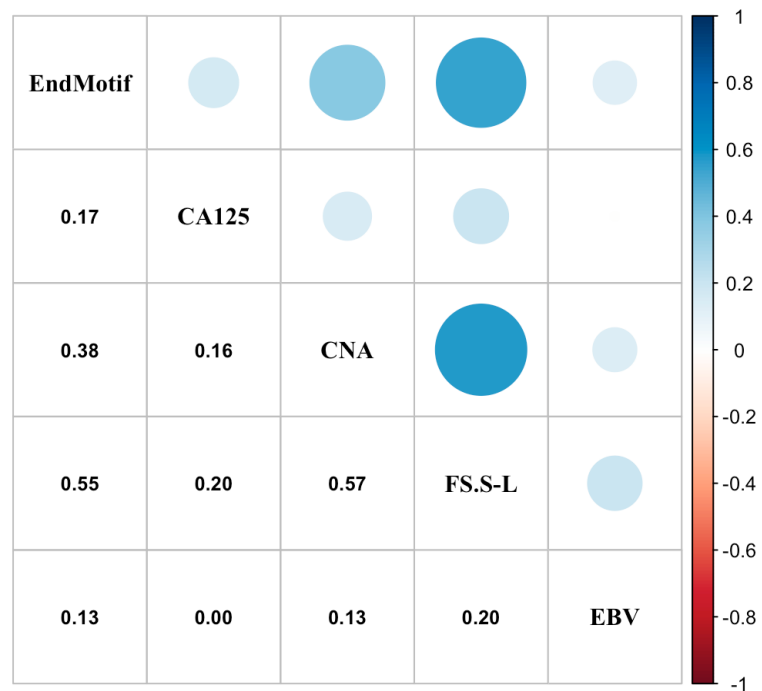

**B**

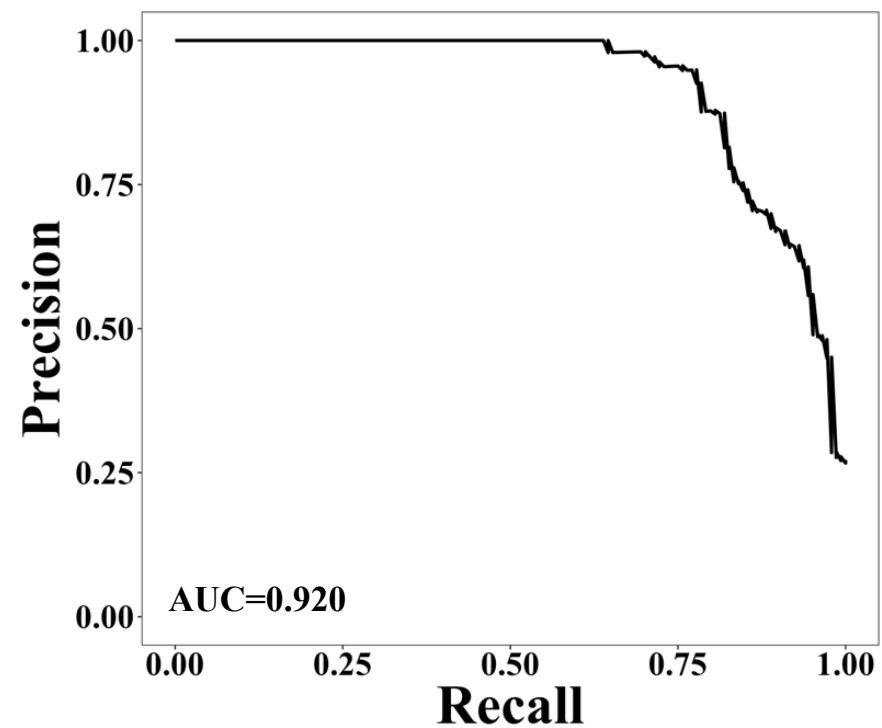

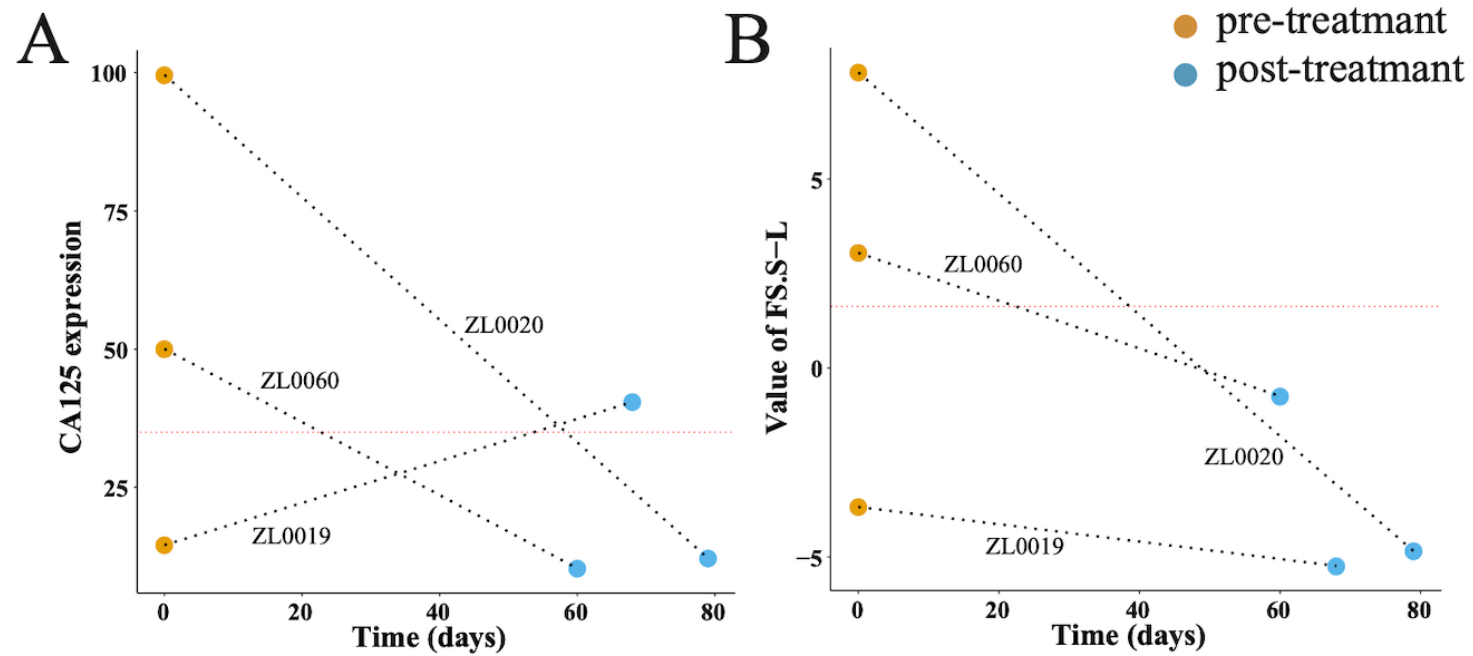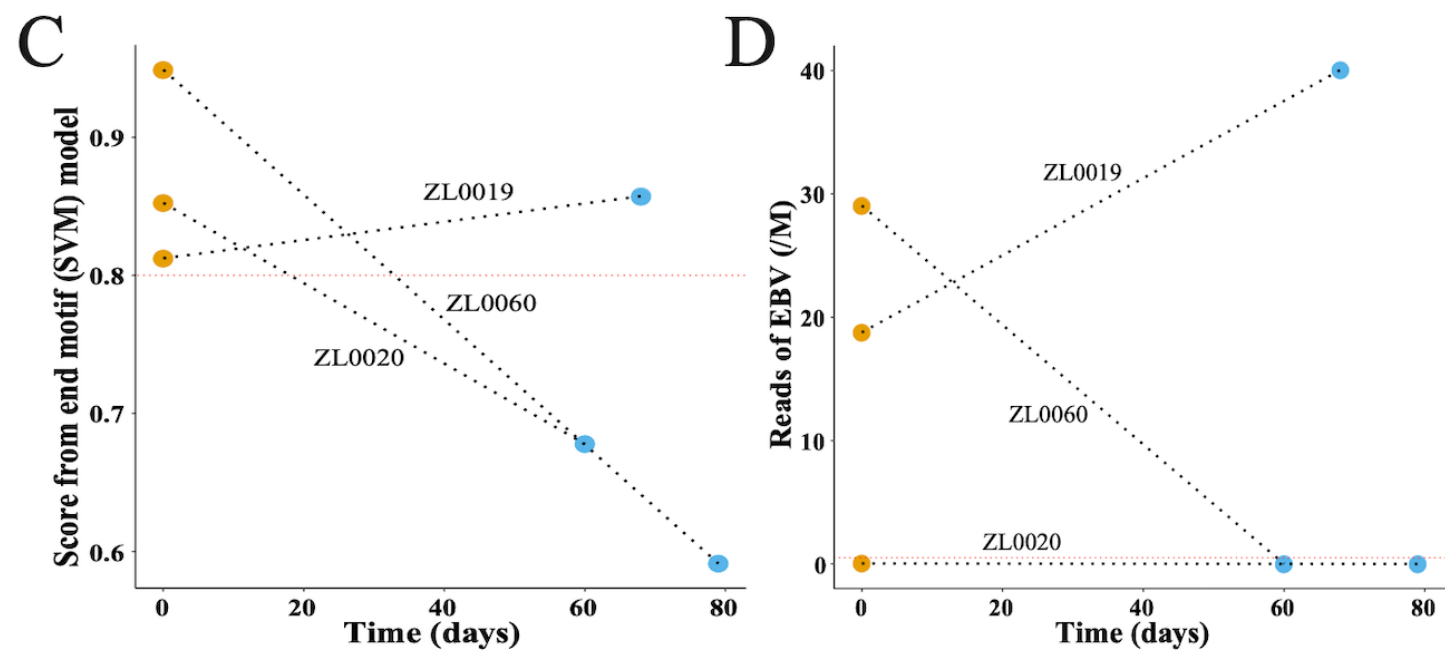

Supplement: Supplementary Figure 1 — Schematic of multi-omics and multidimensional SeekInCare approach. One tube (8ml) of peripheral blood was collected from healthy individuals and lymphoma patients. cfDNA was extracted and then analyzed the cancer genomics, epigenetics, and virus infection through shallow whole genome sequencing. Meanwhile, the expression of seven protein tumor markers was also measured in the plasma. Cancer risk score (CRS) was calculated by machine learning method, which integrated CNA, FS, end motif, virus, and protein marker. CNA, copy number aberration, WGS, whole genome sequencing. [file DataSheet_1.pdf]
